# Dentomaxillofacial abnormalities associated with rare bone disease in two pediatric populations from southern Europe and East Africa

**DOI:** 10.1186/s13023-025-04106-3

**Published:** 2025-11-24

**Authors:** Lluís Brunet-Llobet, Elias Isaack Mashala, Anastasiya Lapitskaya, Judit Rabassa-Blanco, María Dolores Rocha-Eiroa, Jaume Miranda-Rius

**Affiliations:** 1 Department of Dentistry, Hospital Sant Joan de Déu, University of Barcelona, Barcelona, Spain; 2 Department of Odontostomatology, Faculty of Medicine and Health Sciences, University of Barcelona, Barcelona, Spain; 3 Hospital Dentistry and Periodontal Medicine Research Group, Institut de Recerca Sant Joan de Déu (IRSJD), Barcelona, Spain; 4 Department of Orthopedic Surgery and Traumatology, Mount Meru Regional Referral Hospital, Arusha, Tanzania; 5 Doctoral programme in Medicine and Translational Research, Line of Odontostomatology, Faculty of Medicine and Health Sciences, Hospital Sant Joan de Déu, University of Barcelona, Barcelona, Spain

**Keywords:** Dentomaxillofacial disorders, Bone diseases, Congenital condition, Rare diseases, Dental abnormalities, Fluorosis

## Abstract

**Background:**

It is well known that certain bone diseases of congenital origin are associated with dentomaxillofacial (DOMF) disorders. The objective of this study was to evaluate and compare the DOMF alterations in pediatric patients with bone diseases in Arusha (Tanzania, East Africa) and Barcelona (Spain, southern Europe). In each area of study, the clinical differences between subgroups of bone diseases in relation to their etiopathogenesis were reported and analysed.

**Material & methods:**

A cross-sectional study of pediatric patients with bone diseases was carried out at two hospitals, Mount Meru Regional Referral Hospital (MMRRH), Arusha (*n* = 60) and Hospital Sant Joan de Déu (HSJD), Barcelona (*n* = 89), from 2019 to 2023. Mean age of the sample was 10.5 years (SD 4.05). In both groups the samples were recruited consecutively and were clinically evaluated for skeletal and DOMF disorders. The different bone pathologies were further divided into two subgroups according to their etiopathogenesis: (i) disorders in cellular metabolism (DCM); (ii) disorders of bone growth/deformity (DGD).

**Results:**

Gingival health indexes were significantly better in the HSJD group (*p* = 0.033). The HSJD group also had better caries indices (DMF-T), though these differences were not significant (*p* = 0.105). Among dental alterations, dental agenesis was significantly more prevalent in the MMRRH sample (*p* < 0.001); in this sample, DGD was significantly more frequent than DCM (*p* = 0.045). Fluorosis was practically non-existent in the HSJD group, but was moderate to severe fluorosis in 26.6% of MMRRH patients and was significantly more prevalent in the MMRRH DCM subgroup (*p* < 0.001). Malocclusion was more frequent in the MMRRH group (*p* < 0.001 in the case of Class III inverted overjet and *p* = 0.008 in the case of crossbite), and in the HSJD group the DCM subgroup presented a more severe overbite and open bite than the DGD subgroup (*p* = 0.027). Pathological fractures were significantly more frequent in the DGD subgroups in both samples (*p* < 0.001).

**Conclusion:**

There is a clear relationship between dentomaxillofacial abnormalities and rare bone diseases in the two pediatric populations studied. Comparing the two samples, the East African children displayed higher rates of gingivitis, dental fluorosis and malocclusion than their southern European peers.

## Background

 In the European Union, a rare disease is defined as one effecting fewer than five out of 10,000 members of the general population. Approximately 80% of rare diseases are genetic in nature (Directive No 2011/24/EU). The European Reference Network of Rare Bone Diseases (ERN BOND) records all rare diseases, essentially congenital, chronic and of genetic origin, that affect cartilage, bones and dentine [1]. The eleventh version of the Nosology of Genetic Skeletal Disorders, revised in 2023, comprises 771 different diseases associated with 552 genes that are classified into 41 groups based on their clinical, radiographic, and/or molecular phenotypes [2].

Dentomaxillofacial (DOMF) disorders comprise a set of dental abnormalities, periodontal conditions and occlusal disorders that affect the oromaxillofacial region as a result of pre-existing skeletal pathology [3]. Reports in the literature have shown that genetic defects and environmental factors play a role in the etiopathogenesis and development of these diseases. The variability in their clinical presentation has also been reported to contribute to the contrasting severity of DOMF disorders in individuals with bone disease [4–6].

In an effort to establish a clear, clinically oriented relationship between the ‘meeting point’ of bones and teeth, four areas of cellular events and molecular signalling pathways have been identified. (i) The similarity in the interaction of ectoderm and mesoderm gives rise to the common precursor cells (stem cells); ectomesenchyme cells explain the origin and development of both teeth and bones in oromaxillofacial regions [5–13]. (ii) The comparable capacity of ectomesenchyme cells to differentiate into various cell lines: odontoblasts, osteoblasts, fibroblasts and chondrocytes [13–15]. (iii) Similar molecular signaling pathways (growth factors) involved in both odontogenesis and osteogenesis and skeletal development; these include Wnt, Shh, FGF, BMPs, and they have a significant influence in regulations of different stages in both teeth and bones formations. (iv) Similar mineralization processes of the bone matrix, dental matrix and enamel matrix involving hydroxyapatite, mucopolysaccharides, collagen, sialoproteins, calcium and other inorganic substances [16–20].

As the diagnosis of DOMF disorders in children with rare bone disease is frequently overlooked, these conditions are often left untreated. Little information is available on the clinical dental manifestations in these patients or on the diagnostic strategies applied by pediatricians and orthopedic surgeons. Some authors have investigated the possible relationship between rare bone disease and DOMF alterations, but most of these publications have addressed a single entity (e.g., osteogenesis imperfecta, mucopolysaccharidosis, etc.) [7, 11, 12, 21].

The objective of this cross-sectional study was to describe and compare the DOMF disorders presented by pediatric patients with different rare bone diseases from two geographical areas: Arusha, in Tanzania, East Africa, and Barcelona, in southern Europe.

## Materials and methods

A cross-sectional study designed in compliance with the STROBE checklist was performed at Mount Meru Regional Referral Hospital (MMRRH) in Tanzania, East Africa and at the Sant Joan de Déu University teaching hospital (HSJD) in Barcelona, Spain, between 2020 and 2023. The patients with bone pathologies from these two hospitals were investigated for DOMF disorders.

This study was part of a non-profit project approved in advance by the health authorities of Arusha City Council and the ethical committees of the HSJD and the University of Barcelona. Permission was obtained from the regional health authorities to conduct the study, and ethical clearance certificates Ref. no. CD / E.10/39/131 and PIC-175-19 were acquired. The parents of patients in both groups gave informed consent.

### Sample size

The sample of patients with rare bone pathologies from the MMRRH in Arusha (MMRRH-group, *n* = 60) were included as cases in a previous case-control study of maxillofacial disorders in the same geographical area [22]. The sample from Barcelona (HSJD-group, *n* = 89) was calculated based on the frequency of visits to the orthopedic outpatient clinic and on their ability to undergo clinical dentistry at the clinic. The size of the two samples made it possible to achieve a statistical power of 80% for the detection of effect sizes of 0.5 (Cohen’s d) in two-tailed comparisons of the means of the two groups, working with a significance level of 0.05 or less.

### Study area

The epidemiological characteristics of both groups were representative and heterogeneous. Hospital Sant Joan de Déu is a university teaching and pediatric referral hospital in the city of Barcelona, Spain, with 4.4 million inhabitants (2020) and an estimated GDP per capita of $44,300. Mount Meru Regional Referral Hospital is a public regional referral hospital located in the city of Arusha with a catchment area of approximately two million people. The World Bank classifies the United Republic of Tanzania as a lower-middle income economy. Arusha and its surroundings have an average annual per capita income of US$1,185 [7].

### Inclusion and exclusion criteria

Patients aged between 6 and 18 years with rare bone disease, stable mental state and able to cooperate in dental or clinical assessment were included after their parents or caregivers had signed the consent forms. Patients with bone diseases related to trauma, infections and intellectual disability were excluded. The consent forms were provided in their mother tongue languages such as Swahili or English at the MMRRH, and Spanish or English at the HSJD Barcelona Spain. Ethical clearances had been obtained for both samples. Patients with bone diseases related to trauma, infections and intellectual disability were excluded.

### Measurements

Convenience sampling was used to identify patients with bone pathology and to define them as the MMRRH and HSJD groups. Clinical examination of their musculoskeletal system was performed, and data on age, height, weight, skull, spine, and limbs were recorded. Radiological imaging (predominantly X-rays) of the affected part of the skeleton were performed. No additional imaging (for example, CT scan or MRI) or laboratory tests were performed.

Due to the absence of molecular, genetic and chromosomal laboratory studies in the MMRRH group, none of the patients in this group underwent tests to confirm the genetic diagnosis. Diagnosis was therefore provisionally clinical and was based on thorough clinical reports and X-ray results recorded by two consultants, an orthopedic surgeon and a pediatrician. The HSJD group were diagnosed clinically and were confirmed using readily available genetic and chromosomal tests. Additional imaging such as CT-scan and MRI findings were present in the patient’s clinical information system, but this information was excluded from the study.

Skeletal deformities such as malformations of the upper or lower limbs were classified anatomically according to the International Classification of Diseases, Ninth Revision (ICD9) codes 755.2 to 755.4 (for upper, lower, and unspecified limb defects) [23].

In the clinical oral examination of both groups, indices such as the Silness & Löe Plaque Index (PI) were used to assess oral hygiene, and the gums were assessed using the Löe & Silness Gingival Index (GI) [24, 25]. The Decayed, Missing, Filled Teeth (DMF-T) index was used to determine the prevalence of caries and the need for dental treatment [26], and the Thylstrup-Fejerskov (TF) index classified individuals into categories according to the degree of fluorosis: mild (TFI = 1–3), moderate (TFI = 4–5) and severe (TFI = 6–9) [27]. Clinical dental anomalies (dental agenesis, supernumerary teeth, anomalies of shape and position) were recorded and, where possible, a panoramic radiograph was requested. Tooth absences were recorded if one or more teeth were missing from the mouth. For malocclusions, angle classes were recorded as either overjet, overbite or crossbite [28].

The diagnosis of bone pathology was indicated in patients’ clinical records at both institutions. The dental measurement system was calibrated prior to data recording. The three examiners (JMR, LBL and EIM) received joint training in the evaluation of dentomaxillofacial abnormalities and assessed several patients using the established criteria. The results were then compared to identify discrepancies and reach a consensus. Following advice from the two most senior practitioners (JMR and LBL), the three examiners adjusted their assessments several times until maximum consistency was achieved. The concordance of the measurements was assessed using the Kappa index, with a 95% confidence interval, which was found to be 0.84.

### Data interpretation and statistical analysis

Several pathologies were difficult to compare due to the small number of patients affected. Mashala et al.’s classification method according to etiopathogenesis was used to group the different entities into two subgroups of cases for intra-group comparison [22]. The different bone pathologies were divided into two subgroups: (i) Disorders in Cellular Metabolism (DCM); (ii) Disorders of bone Growth/Deformity (DGD). Musculoskeletal and clinical examinations were performed in both groups.

A descriptive analysis was performed of the two groups and subgroups studied. Frequencies were calculated for categorical variables and corresponding descriptive statistics for numerical variables. QQ plots were used to assess the normality of the distribution of numerical variables.

For categorical variables, the chi-squared test or Fisher exact test was used to assess differences between the groups and between different bone pathologies. The Student’s t-test or the Mann-Whitney U test (depending on the distribution of the variable) was used for numerical variables. P-values of 0.05 or less were considered as significant. The analysis was performed using the Statistical Package for the Social Sciences (SPSS for Windows, version 23.00 IBM Inc, Amonk, NY, USA).

## Results

The total sample comprised 149 patients with bone diseases (MMRRH: *n* = 60, 40.3%, HSJD: *n* = 89, 59.7%). Almost half of the patients (*n* = 83, 55.7%) were of European origin, 63 were African (42.3%), one American (0.7%) and two Asian (1.3%). With regard to bone pathology, the DCM subgroup comprised 70 patients (47%) and the DGD subgroup 79 patients (53%). In the DCM subgroup, mucopolysaccharidosis was the most common bone pathology in the HSJD sample (*n* = 25, 28.1%), while in the MMRRH sample the most frequent conditions were congenital limb bone deficiencies (*n* = 11, 34.4%) followed by neural tube defect (*n* = 10, 31.3%). The most common bone pathology in the DGD subgroup in the HSJD sample was osteogenesis imperfecta (*n* = 20, 22.5%) followed by achondroplasia (*n* = 17, 19.1%); in the MMRRH sample it was also osteogenesis imperfecta (*n* = 11, 39.3%) followed in this case by arthrogryposis (*n* = 9, 32.1%) (Table 1).

**Table 1:** Bone disease pathology

Bone disease group	MMRRH group (*N* = 60)		HSJD group (*N* = 89)
	Differential diagnosis	Frequency (%)	Frequency (%)
Disorders in cellular metabolism (DCM): *N* = 70	Fibrous dysplasia	2 (6.2%)	4(4.5%)
	Hypophosphatemic rickets	5 (15.6%)	4(4.5%)
	Craniosynostosis	3 (9.4%)	4(4.5%)
	Mucopolysaccharidosis	2 (6.2%)	25(28.1%)
	Cong limb bone deficiencies	11 (34.4%)	1(1.1%)
	NTD - Spina bifida	10 (31.3%)	0(0.0%)
	***Total***	***32 (53.3%)***	***38(42.7%)***
Disorders of bone Growth/deformity (DGD): *N* = 79	Osteogenesis imperfecta	11 (39.3%)	20(22.5%)
	Neurofibromatosis	3 (10.7%)	2(2.2%)
	Arthrogryposis	9 (32.1%)	1(1.1%)
	Dysplasia	2 (7.1%)	11(12.4%)
	Achondroplasia	3(10.7%)	17(19.1)
	**Total**	**28 (46.7%)**	**51(57.3%)**

Percentages for each differential diagnosis are calculated regarding the total number of cases for the corresponding bone disease group and hospital

Percentages for the total diagnosis are calculated regarding the total number of cases for the corresponding hospital

The mean age of the overall sample (*n* = 149) was 10.5 years (SD 4.05) with no differences between the two populations analysed (*p* = 0.882) (Table 2a). As for gender distribution, most patients were male (*n* = 81, 54.4%); the difference between geographical groups was not significant (*p* = 0.868) nor were there significant differences between the DCM and DGD subgroups at the MMRRH (*p* = 0.128) or at the HSJD (*p* = 0.691) (Table 2a, b).

**Table 2a:** Sociodemographic data and oral manifestations (overall and per hospital)

Variable:	All data (*N* = 149)	Hospital comparison		
		HSJD Group (*N* = 89)	MMRRH group (*N* = 60)	*p*-value
Age: (years)	10.51 (4.05)	10.47 (4.25)	10.57 (3.76)	0.882 ^(1)^
**Sex**: Female Male	68 (45.6%) 81 (54.4%)	40 (44.9%) 49 (55.1%)	28 (46.7%) 32 (53.3%)	0.868 ^(3)^
Mean height (cm); (SD)	124.31 (22.04)	124.20(22.359)	124.42(21.88)	0.957 ^(1)^
Mean weight: (kg)	34.43 (14.70)	33.87(16.27)	35.07(12.79)	0.214 ^(2)^
Mean Mouth opening (cm); (SD)	37.0 (5.65)	34.43(3.73)	41.0(6.49)	**< 0.001** *^(2)^
**Dentition**:				
Temporary Mixed Permanent	23 (15.4%) 75 (50.3%) 51 (34.2%)	13(14.6%) 51(57.3%) 25 (28.1%)	10(16.7%) 24(40.0%) 26(43.3%)	0.97^(3)^
**Plaque Index**:				
PI 0 PI 1 PI 2 PI 3	15 (10.1%) 58 (38.9%) 54 (36.2%) 22 (14.8%)	9 (10.1%) 26 (29.2%) 39 (43.8%) 15 (16.9%)	6 (10.0%) 32 (53.3%) 15 (25.0%) 7 (11.7%)	0.023* ^(2)^
**Gingival index**:				
GI 0 GI 1 GI 2 GI 3	3 (2.0%) 29 (19.5%) 81 (54.4%) 36 (24.2%)	3 (3.4%) 23 (25.8%) 42 (47.2%) 21(23.6%)	0 6 (10.0%) 39 (65.0%) 15 (25.0%)	0.033* ^(2)^
**DMF-T**:				
0 1–2 > 2	96 (64.4%) 21 (14.1%) 32 (21.5%)	62 (69.7%) 10 (11.2%) 17 (19.1%)	34 (56.7%) 11 (18.3%) 15 (25.0%)	0.105 ^(2)^
**Dental fluorosis**:				
No Mild Moderate Severe	132 (88.6%) 6 (4.0%) 8 (5.4%) 3 (2.0%)	88 (98.9%) 1 (1.1%) - -	44 (73.3%) 5 (8.3%) 8 (13.3%) 3 (5.0%)	0.001* ^(2)^

(*) *p*-value < 0.05: significant. Numbers next to the *p*-value show which test was used: Student’s t-test (1), Mann-Whitney’s U-test (2), chi-square test (3) or Fisher’s exact test (4)

**Table 2b:** Sociodemographic data and oral manifestations (per disease group)

Variable:	All data			HSJD group (*N* = 89)			MMRRH group (*N* = 60)		
	DCM (*N* = 70)	DGD (*N* = 79)	*p*-value	DCM (*N* = 38)	DGD (*N* = 51)	p-value	DCM (*N* = 32)	DGD (*N* = 28)	*p*-value
Age: (years)	11.14 (4.08)	9.94 (3.96)	0.070 ^(1)^	11.21 (4.49)	9.90 (4.02)	0.153 ^(1)^	11.1 (3.6)	10.0 (3.9)	0.279 ^(1)^
**Sex**: Female Male	30 (42.9%) 40 (57.1%)	38 (48.1%) 41 (51.9%)	0.521 ^(3)^	18 (47.4%) 20 (52.6%)	22 (43.1%) 29 (56.9%)	0.691 ^(3)^	12 (37.5%) 20 (62.5%)	16 (57.1%) 12 (42.9%)	0.128 ^(3)^
Mean height (cm) (SD)	128.19 (23.27)	121 (20.54)	0.070 ^(1)^	125.32 (27.91)	123.49 (18.32)	0.773 ^(1)^	130.4 (19.1)	117.5 (23.2)	0.021* ^**(1)**^
Mean weight(kg)	38.28 (16.37)	31.14 (12.30)	0.012* ^(2)^	37.32 (20.12)	31.60 (12.93)	0.390 ^(2)^	39.1 (12.7)	30.5 (11.5)	0.008* ^(2)^
**Dentition**:									
Temporary Mixed Permanent	9 (12.9%) 33 (47.1%) 28 (40.0%)	14 (17.7%) 42 (53.2%) 23 (29.1%)	0.346 ^(3)^	5 (13.2%) 22 (57.9%) 11 (28.9%)	8 (15.7%) 29 (56.9%) 14 (27.5%)	0.943 ^(3)^	4 (12.5%) 11 (34.4%) 17 (53.1%)	6 (21.4%) 13 (46.4%) 9 (32.1%)	0.250 ^(3)^
**Plaque Index**:									
PI 0 PI 1 PI 2 PI 3	7 (10.0%) 25 (35.7%) 28 (40.0%) 10 (14.3%)	8 (10.1%) 33 (41.8%) 26 (32.9%) 12 (15.2%)	0.636 ^(2)^	6 (15.8%) 8 (21.1%) 18 (47.4%) 6 (15.8%)	3 (5.9%) 18 (35.3%) 21 (41.2%) 9 (17.6%)	0.874 ^(2)^	1 (3.1%) 17 (53.1%) 10 (31.3%) 4 (12.5%)	5 (17.9%) 15 (53.6%) 5 (17.9%) 3 (10.7%)	0.111 ^(2)^
**Gingival index**:									
GI 0 GI 1 GI 2 GI 3	2 (2.9%) 11 (15.7%) 38 (54.3%) 19 (27.1%)	1 (1.3%) 18 (22.8%) 43 (54.4%) 17 (21.5%)	0.345 ^(2)^	2 (5.3%) 10 (26.3%) 17 (44.7%) 9 (23.7%)	1 (2.0%) 13 (25.5%) 25 (49.0%) 12 (23.5%)	0.738 ^(2)^	0 1 (3.1%) 21 (65.6%) 10 (31.3%)	0 5 (17.9%) 18 (64.2%) 5 (17.9%)	0.069 ^(2)^
**DMF-T**:									
0 1–2 > 2	49 (70.0%) 7 (10.0%) 14 (20.0%)	47 (59.5%) 14 (17.7%) 18 (22.8%)	0.291 ^(2)^	27 (71.1%) 4 (10.5%) 7 (18.6%)	35 (68.6%) 6 (11.8%) 10 (19.6%)	0.759 ^(2)^	22 (68.8%) 3 (9.4%) 7 (21.8%)	12 (42.8%) 8 (28.6%) 8 (28.6%)	0.166 ^(2)^
**Dental Fluorosis**									
No Mild Moderate Severe	56 (80.0%) 5 (7.1%) 6 (8.6%) 3 (4.3%)	76 (96.2%) 1 (1.3%) 2 (2.5%) -	0.768 ^(2)^	38 (100%) - - -	50 (98.0%) 1 (2.0%) - -	-	0 (80.0%) 5 (7.1%) 6 (8.6%) 3 (4.3%)	0 (96.2%) 1 (1.3%) 2 (2.5%) -	0.001* ^(2)^

(*) *p*-value < 0.05: significant. Numbers next to the *p*-value show which test was used: Student’s t-test (1), Mann-Whitney’s U-test (2), chi-square test (3) or Fisher’s exact test (4)

The mean height of the patients across the study was 124.31 cm (SD 22.04). At the MMRRH, patients in the DCM subgroup were taller (130.4 cm, SD 19.1) than those in the DGD subgroup (117.5 cm, SD 23.2), the difference being significant (*p* = 0.021). At the HSJD there was no difference in mean height between the DCM and DGD subgroups (*p* = 0.773) (Table 2a, b). The mean weight was 34.43 kg (SD 14.7), with no difference between MMRRH and HSJD groups (*p* = 0.214), though in the MMRRH sample the mean weight differed significantly (*p* = 0.008) between the DCM (39.1 kg, SD 12.7) and DGD (30.5 kg, SD 11.5) subgroups; a similar trend was observed at the HSJD, but the differences were not significant (*p* = 0.390) (Table 2a, b).

Oral examination revealed mixed dentition in 50.3% of the sample, with a similar distribution for both populations analysed. Moderate and severe gingival inflammation was observed in the entire study population, with a prevalence of 78.6% (GI 2 & 3). Gingival indices were higher in the MMRRH sample than in the HSJD group, and these differences were significant (*p* = 0.033). There was no significant difference between the DCM and DGD subgroups in either sample (Table 2a, b).

The prevalence of plaque was high at both hospitals (51% PI 2 & 3). The HSJD group had a significantly higher rate of PI (60.7%) than the MMRRH group (36.7%) (*p* = 0.023). However, no significant difference was observed between DCM and DGD subgroups at either hospital (Table 2a, b).

Almost two-thirds of the whole population (64.4%) had a DMF-T index grade 0, with no significant differences between the two hospitals (*p* = 0.105). The prevalence of mild to severe dental fluorosis in the MMRRH sample was 26.7% compared with 1.1% in the HSJD sample, this difference being clearly significant (*p* = 0.001). Among MMRRH subjects, DCM (20%) was more prevalent than DGD (3.8%), and again the difference was significant (*p* = 0.001) (Table 2a, b).

The mean mouth opening for all samples was 37 cm (SD 5.65). Subjects from the MMRRH reported a mean mouth opening of 41 cm (SD = 6.49), significantly greater than in the HSJD subjects (34.43 cm, SD = 3.73) (*p* < 0.001) (Table 2a, b).

The following dental abnormalities were recorded: shape, supernumerary teeth, clinical absence of teeth (dental agenesis) and position. Clinical absence of teeth (dental agenesis) was more prevalent in the MMRRH sample (*n* = 22, 36.7%) than in the HSJD sample (*n* = 9, 10.1%), the difference being significant (*p* < 0.001). Furthermore, this abnormality in the MMRRH was found to be significantly more prevalent in the DGD (*n* = 14, 50%) than in the DCM (*n* = 8, 25%) subgroups (*p* = 0.045). Other variables such as clinical supernumerary, shape anomaly and positional anomaly did not differ significantly between hospitals, nor between DCM or DGD subgroups at each hospital (Table 3).

**Table 3:** Dental abnormalities and occlusion

Variable	HSJD group (*N* = 89)	MMRRH group (*N* = 60)	*p*-value	HSJD group (*N* = 89)			MMRRH group (*N* = 60)		
Dental abnormalities				DCM (*N* = 38)	DGD (*N* = 51)	*p*-value	DCM (*N* = 32)	DGD (*N* = 28)	*p*-value
Clinical Supernumerary teeth:	12(13.5%)	5 (8.3%)	0.332 ^(3)^	7 (18.4%)	5 (9.8%)	0.239 ^(3)^	2 (6.3%)	3 (10.7%)	0.657 ^(4)^
Shape abnormalities:	6(6.7%)	9 (15.0%)	0.100 ^(3)^	2 (5.3%)	4 (7.8%)	1.000 ^(4)^	5 (15.6%)	4 (14.3%)	1.000 ^(4)^
Position abnormalities:	13(14.6%)	13 (21.7%)	0.265 ^(3)^	6 (15.8%)	7 (13.7%)	0.785 ^(3)^	9 (28.1%)	4 (14.3%)	0.226 ^(4)^
Clinical absence of teeth:	9(10.1%)	22 (36.7%)	< 0.001* ^(3)^	4 (10.5%)	5 (9.8%)	1.000 ^(4)^	8 (25.0%)	14 (50.0%)	0.045* ^(3)^
**Occlusion**:									
Class I Class II Class III	50 (56.2%) 29 (32.5%) 10 (11.2%)	41 (68.3%) 16 (26.7%) 3 (5.0%)	0.105 ^(2)^	19 (50.0%) 16 (42.1%) 3 (7.9%)	31 (60.8%) 13 (25.5%) 7 (13.7%)	0.525 ^(2)^	19 (59.4%) 10 (31.2%) 3 (9.4%)	22 (78.6%) 6 (21.4%) 0	0.083 ^(2)^
**Overjet**:									
Inverted (< 0 mm) Edge to edge (0–1 mm) Normal (2–3 mm) Increased (> 3 mm)	7 (8.0%) 10 (11.4%) 46 (52.3%) 25 (28.4%)	5 (8.3%) 36 (60.0%) 14 (23.4%) 5 (5.0%)	< 0.001* ^(2)^	1 (2.7%) 4 (10.8%) 17 (45.9%) 15 (40.5%)	6 (11.8%) 6 (11.8%) 29 (56.9%) 10 (19.6%)	0.023* ^(2)^	3 (9.4%) 16 (50.0%) 9 (28.1%) 4 (12.5%)	2 (7.1%) 20 (71.4%) 5 (17.9%) 1 (3.6%)	0.703 ^(2)^
**Overbite**:									
Open bite (< 0 mm) Edge to edge (0–1 mm) Normal (2–3 mm) Increased (> 3 mm)	26 (29.5%) 11 (12.5%) 32 (36.4%) 19 (21.6%)	3 (5.0%) 36 (60.0%) 17 (28.3%) 4 (6.7%)	< 0.001* ^(2)^	15 (40.5%) 6 (16.2%) 11 (29.7%) 5 (13.5%)	11 (21.6%) 5 (9.8%) 21 (41.2%) 14 (27.5%)	0.027* ^(2)^	3 (9.4%) 15 (46.8%) 11 (34.4%) 3 (9.4%)	0 21 (75.0%) 6 (21.4%) 1 (3.6%)	0.239 ^(2)^
**Crossbite**:									
No Yes	81 (92.0%) 7 (8.0%)	46 (76.7%) 14 (23.3%)	0.008* ^(3)^	35 (94.6%) 2 (5.4%)	46 (90.2%) 5 (9.8%)	0.694 ^(4)^	29 (90.6%) 3 (9.4%)	28 (100%) 0 (0.0%)	0.241 ^(4)^

(*) *p*-value < 0.05: significant. Numbers next to the *p*-value show which test was used: Student’s t-test (1), Mann-Whitney’s U-test (2), chi-square test (3) or Fisher’s exact test (4)

With regard to the type of dental occlusion, the HSJD sample presented more Angle Class II (*n* = 29, 32.5%) and Class III (*n* = 10, 11.2%) than the MMRRH sample (*n* = 16, 26.7% and *n* = 3, 5.0% respectively); however, the difference was not significant (*p* = 0.105). No differences were observed between the DCM and DGD subgroups in either hospital (Table 3).

In relation to overjet, the MMRRH group demonstrated a clear propensity for exhibiting edge-to-edge or inverted incisors (*n* = 41, 68.3%). In contrast, overjet values exceeding 3 mm were more frequent in the HSJD group (*n* = 25, 28.4%), and this disparity was statistically significant (*p* < 0.001). At the HSJD, overjet greater than 3 mm was clearly more prevalent in the DCM subgroup (40.5%) than in the DGD subgroup (19.6%), the difference being significant (*p* = 0.023). With regard to overbite, open bite was more prevalent at the HSJD than at the MMRRH (*p* < 0.001); at the HSJD, the difference between the DCM subgroup (40.5%) and the DGD subgroup (21.6%) was significant (*p* = 0.027). A significant tendency toward crossbite occlusion was evident at the MMRRH (23.3%) but not at the HSJD (8.0%) (*p* = 0.008) (Table 3).

As outlined in Table 4, the most common skeletal manifestations in both hospitals were lower limb deformities (85.0% at MMRRH and 65.2% at HSJD). Similar rates were seen for pathological fractures (26.7% and 24.7%, respectively). In both hospitals, the DGD subgroup had a significantly higher incidence of pathological fractures than the DCM subgroup (*p* < 0.001) (Table 4).

**Table 4:** Skeletal manifestation

Skeletal manifestation	MMRRH group (*N* = 60)				HSJD group (*N* = 89)			
	DCM (*N* = 32)	DGD (*N* = 28)	TOTAL(*N* = 60)	*p*-value	DCM (*N* = 38)	DGD(*N* = 51)	TOTAL (*N* = 89)	*p*-value
Skull deformities	5 (15.6%)	3 (10.7%)	8(13.3%)	0.712 ^(4)^	3 (7.9%)	5 (9.8%)	8(9.0%)	1.000 ^(4)^
Facial shape deformities	2 (6.3%)	6 (21.4%)	8(13.3%)	0.130 ^(4)^	2 (5.3%)	2 (3.9%)	4(4.5%)	1.000 ^(4)^
Cranial flattening	2 (6.3%)	4 (14.3%)	6(10.0%)	0.404 ^(4)^	1 (2.6%)	1 (2.0%)	2(2.2%)	1.000 ^(4)^
Early craniosynostosis	4 (12.5%)	2 (7.1%)	6(10.0%)	0.675 ^(4)^	----	1 (2.0%)	1(1.1%)	1.000 ^(4)^
Spine deformities	7 (21.9%)	12 (42.9%)	19(31.7%)	0.081 ^(3)^	19 (50.0%)	29 (56.9%)	48(53.9%)	0.521 ^(3)^
Upper limb deformities	8 (25.0%)	6 (21.4%)	14(23.3%)	0.391 ^(3)^	17 (44.7%)	17 (33.3%)	33(37.1%)	0.273 ^(3)^
Lower limb deformities	26 (81.3%)	25 (89.3%)	51(85.0%)	0.482 ^(3)^	24 (63.2%)	34 (66.7%)	58(65.2%)	0.731 ^(3)^
Pathological fractures	2 (6.3%)	14 (50.0%)	16(26.7%)	< 0.001* ^(4)^	2 (5.3%)	20 (39.2%)	22(24.7%)	< 0.001* ^**(**4)^

(*) *p*-value < 0.05: significant. Numbers next to the *p*-value show which test was used: chi-square test (3) or Fisher’s exact test (4)

Note: It must be considered that the same patient may present more than one skeletal disorder

The prevalence of upper limb deformity was 37.1% at the HSJD and 23.3% at the MMRRH, the difference not being significant (*p* = 0.112). Furthermore, all participants of the DGD subgroup exhibited intercalary and longitudinal defects of the arm and forearm (Table 5). The findings indicated a high prevalence of lower limb deformities (85.0% at the MMRRH and 65.2% at the HSJD), the difference being significant (*p* = 0.013). In terms of other patterns of lower limb malformations, the DCM and DGD subgroups at both hospitals had comparable frequencies of multiple lower limb malformations, various knee deformities and clubfoot (Table 5).

**Table 5:** Upper and lower limb deformities

Anatomical classification^#^	Specific anatomical location	MMRRH group (*N* = 60)			HSJD group (*N* = 89)			
		DCM (*N* = 32)	DGD (*N* = 28)	TOTAL (*N* = 60)	DCM (*N* = 38)	DGD (*N* = 51)	TOTAL (*N* = 89)	*p*-value
**Upper limb** **deformities**								
Intercalary	Forearm	-	2 (33.3%)	2 (14.3%)	2 (12.5%)	3 (17.6%)	5 (15.2%)	
	Arm	-	-	-	1 (6.3%)	4 (23.5%)	5 (15.2%)	
Longitudinal	Arm and forearm	-	4(66.7%)	4 (28.6%)	-	4 (23.5%)	4 (12.1%)	
Terminal transverse	Below elbow	3(37.5%)	-	3 (21.4%)	3 (18.8%)	1 (5.9%)	4 (12.1%)	
	MCP (fingers)	1(12.5%)	-	1 (7.1%)	1 (6.3%)	-	1 (3.0%)	
Congenital absence	Congenital absence	2(25.0%)	-	2 (14.3%)	-	-	-	
Club hand	Club hand	2(25.0%)	-	2 (14.3%)	6 (37.5%)	3 (17.6%)	9 (27.2%)	
Multiple deformities	Multiple deformities	-	-	-	3 (18.8%)	2(11.8%)	5 (15.2%)	
**TOTAL**		8 (25.0%)	6 (21.4%)	14 (23.3%)	16 (42.1%)	17 (33.3%)	33 (37.1%)	0.112 ^(3)^
**Lower limb** **deformities**								
Intercalary	Femur/thigh	2 (7.7%)	-	2 (3.9%)	1 (4.2%)	3 (9.4%)	4(4.5%)	
Longitudinal	Tibia/fibula	1 (3.8%)	6 (24.0%)	7 (13.7%)	1 (4.2%)	5 (15.6%)	6(6.7%)	
Terminal transverse	Above knee (upper leg)	-	3(12.0%)	3 (5.9%)	6 (25.0%)	3 (9.4%)	9(10.1%)	
	Below knee (lower leg)	9 (34.6%)	4(16.0%)	13 (25.5%)	3 (12.5%)	2 (6.3%)	5(5.6%)	
	MTP (toes)	2 (7.7%)	0(0.0%)	2 (3.9%)	-	-	0(0.0%)	
Multiple deformities	Multiple deformities	4(15.4%)	5(20.0%)	9 (17.6%)	2 (8.3%)	12 (37.5%)	14(15.7%)	
Congenital absence	Congenital absence	2(7.7%)	-	2 (3.9%)	-	-	0(0.0%)	
**Miscellaneous bone deformity**								
Hip	Hip dysplasia	-	2(8.0%)	2 (3.9%)	1 (4.2%)	1 (3.1%)	2(2.2%)	
Knee	Knee joint deformity	4(15.4%)	3(12.0%)	7 (13.7%)	8 (33.3%)	6 (18.8%)	14(15.7%)	
Foot	Club foot	2(7.7%)	2 (8.0%0)	4 (7.8%)	2 (8.3%)	-	2(2.2%)	
**TOTAL**		26(81.3%)	25(89.3%)	51(85%)	24 (63.2%)	32 (62.7%)	58(65.2%)	**0.013** ^* (3)^

**#** Anatomical classification used by the International Classification of Diseases, Ninth Revision (ICD9) codes 755.2 to 755.4

(For upper, lower and unspecified limb defects). Two cases in the DCM have non-specific upper limb deformities

(*) *p*-value < 0.05: significant. Numbers next to the *p*-value show which test was used: chi-square test (3) or Fisher’s exact test (4)

No association was found between malocclusion and skeletal deformities (spine and upper limb) in the population as a whole, or in the HSJD sample. In contrast, a significant correlation was identified in the MMRRH sample between upper and lower limb deformities and inverted or edge-to-edge incisal overjet malocclusion (*p* = 0.031 and *p* = 0.007). A similar correlation was also observed between lower limb deformities and open bite (*p* = 0.002). (Tables 6a, b, c).

**Table 6a:** Relationship between malocclusion and skeletal deformities in overall sample

Variables	Spine Deformity			Upper limb deformity			Lower limb deformity		
Malocclusion	No (*N* = 82)	Yes (*N* = 67)	*p*-value	No (*N* = 99)	Yes (*N* = 50)	*p*-value	No (*N* = 40)	Yes (*N* = 109)	*p*-value
Class I Class II Class III	49 (59.7%) 25 (30.5%) 8 (9.8%)	42 (62.6%) 20 (29.9%) 5 (7.5%)	0.665 ^(2)^	65 (65.6%) 26 (26.3%) 8 (8.1%)	26 (52.0%) 19 (38.0%) 5 (10.0%)	0.127 ^(2)^	25 (62.5%) 13 (32.5%) 2 (5%)	66 (60.6%) 32 (29.4%) 11 (10.1%)	0.686 ^(2)^
Overjet									
Inverted (< 0 mm) Edge to edge (0–1 mm) Normal (2–3 mm) Increased (> 3 mm)	7 (8.6%) 27 (33.3%) 30 (37.1%) 17 (21.0%)	5 (7.5%) 19 (28.4%) 30 (44.7%) 13 (19.4%)	0.725 ^(2)^	5 (5.1%) 33 (33.7%) 41 (41.8%) 19 (19.4%)	7 (14.0%) 13 (26.0%) 19 (38.0%) 11 (22.0%)	0.708 ^(2)^	9 (23.1%) 6 (15.4%) 3 (7.7%) 21 (53.8%)	37 (33.9%) 24 (22%) 9 (8.3%) 39 (35.8%)	0.897 ^(2)^
Overbite									
Open bite (< 0 mm) Edge to edge (0–1 mm) Normal (2–3 mm) Increased (> 3 mm)	15 (18.5%) 26 (32.1%) 28 (34.6%) 12 (14.8%)	14 (20.9%) 21 (31.3%) 21 (31.3%) 23 (15.5%)	0.836 ^(2)^	17 (17.3%) 32 (32.7%) 32 (32.7%) 17 (17.3%)	12 (24.0%) 15 (30.0%) 17 (34.0%) 6 (12.0%)	0.334 ^(2)^	7 (17.9%) 7 (17.9%) 10 (25.6%) 15 (38.5%)	40 (36.7%) 16 (14.7%) 19 (17.4%) 34 (31.2%)	0.444 ^(2)^
Crossbite	9 (11.1%)	12 (17.9%)	0.238 ^(3)^	12 (12.2%)	9 (18.0%)	0.343 ^(3)^	4 (10.3%)	17 (15.6%)	0.594 ^(4)^

(*) *p*-value < 0.05: significant. Numbers next to the *p*-value show which test was used: Student’s t-test (1), Mann-Whitney’s U-test (2), chi-square test (3) or Fisher’s exact test (4)

**Table 6b:** Relationship between malocclusion and skeletal deformities in MMRRH group

Variable	Spinal Deformity			Upper limb deformity			Lower limb deformity		
Malocclusion	No (*N* = 41)	Yes (*N* = 19)	*p*-value	No (*N* = 44)	Yes (*n* = 16)	*p*-value	No (*N* = 9)	Yes (*N* = 51)	*p*-value
Class I Class II Class III	28(68.3%) 10(24.4%) 3(7.3%)	13(66.7%) 6 (33.3%) -	0.861 ^(2)^	32(72.7%) 11(25.0%) 1(2.3%)	9(56.3%) 5(31.2%) 2(12.5%)	0.172 ^(2)^	5 (55.6%) 3 (33.3%) 1 (11.1%)	36 (70.6%) 13 (25.5%) 2 (3.9%)	0.342 ^(2)^
Overjet									
Inverted (< 0 mm) Edge to edge (0–1 mm) Normal (2–3 mm) Increased (> 3 mm)	3(7.3%) 24(58.5%) 10(24.4%) 4(9.8%)	2(10.5%) 12(63.2%) 4(21.1%) 1(5.3%)	0.539 ^(2)^	1(2.3%) 27(61.4%) 12(27.3%) 4(9.0%)	4(25%) 9(56.3%) 2(12.5%) 1(6.2%)	0.031* ^(2)^	7 (77.8%) 0 2 (22.2%) 0	29 (56.9%) 5 (9.8%) 3 (5.9%) 14 (27.5%)	0.007 * ^(2)^
Overbite									
Open bite (< 0 mm) Edge to edge (0–1 mm) Normal (2–3 mm) Increased (> 3 mm)	3(7.3%) 23(56.1%) 12(29.3%) 3(7.3%)	0 13(68.4%) 5(26.3%) 1(5.3%)	0.856 ^(2)^	1(2.3%) 28(63.6%) 12(27.3%) 3(6.8%)	2(12.5%) 8(50.0%) 5(31.3%) 1(6.2%)	0.646 ^(2)^	7 (77.8%) 0 2 (22.2%) 0	29 (56.9%) 4 (7.8%) 1 (2%) 17 (33.3%)	0.002 * ^(2)^
Crossbite	8(19.5%)	6(31.6%)	0.304 ^(3)^	9(20.5%)	5(31.3%)	0.382 ^(3)^	3 (33.3%)	11 (21.6%)	0.423 ^(4)^

(*) *p*-value < 0.05: significant. Numbers next to the *p*-value show which test was used: Student’s t-test (1), Mann-Whitney’s U-test (2), chi-square test (3) or Fisher’s exact test (4)

**Table 6c:** Relationship between malocclusion and skeletal deformities HSJD

Variable	Spinal deformity			Upper limb deformity			Lower limb deformity		
Malocclusion	No (*N* = 41)	Yes (*N* = 48)	*p*-value	No (*N* = 55)	Yes (*N* = 34)	*p*-value	No (*N* = 31)	Yes (*N* = 58)	*p*-value
Class I Class II Class III	21 (51.2%) 15 (36.6%) 5 (12.2%)	29 (60.4%) 14 (29.2%) 5 (10.4%)	0.414^(2)^	33 (60.0%) 15 (27.3%) 7 (12.7%)	17 (50.0%) 14 (41.2%) 10 (11.2%)	0.524^(2)^	20 (64.5%) 10 (32.3%) 1 (3.2%)	30 (51.7%) 19 (32.8%) 9 (15.5%)	0.145^(2)^
Overjet									
Inverted (<0mm) Edge to edge (0-1mm) Normal (2-3mm) Increased (>3mm)	4 (10.0%) 3 (7.5%) 20 (50.0%) 13 (32.5%)	3 (6.3%) 7 (14.6%) 26 (54.2%) 12 (25.0%)	0.506^(2)^	4 (7.4%) 6 (11.1%) 29 (53.7%) 15 (27.8%)	3 (8.8%) 4 (11.8%) 17 (50.0%) 10 (29.4%)	0.955^(2)^	2 (6.7%) 6 (20%) 1 (3.3%) 21 (70%)	8 (13.8%) 19 (32.8%) 6 (10.3%) 25 (43.1%)	0.691^(2)^
Over bite									
Open bite (<0mm) Edge to edge (0-1mm) Normal (2-3mm) Increased (>3mm	12 (30.0%) 3 (7.5%) 16 (40.0%) 9 (22.5%)	14 (29.2%) 8 (16.7%) 16 (33.3%) 10 (20.8%)	0.452^(2)^	16 (29.6%) 4 (7.4%) 20 (37.0%) 14 (25.9%)	10 (29.4%) 7 (20.6%) 12 (35.3%) 5 (14.7%)	0.339^(2)^	0 7 (23.3%) 8 (26.7%) 15 (50%)	11 (19%) 12 (20.7%) 18 (31%) 17 (29.3%)	0.098^(2)^
Crossbite	1 (2.5%)	6 (12.5%)	0.121^(4)^	3 (5.6%)	4 (11.8%)	0.422 ^(4)^	1 (3.3%)	6 (10.3%)	0.414^(4)^

(*) *p*-value < 0.05: significant. Numbers next to the *p*-values show which test was used: Student’s t-test (1), Mann-Whitney’s U-test (2), chi-square test (3) or Fisher’s exact test (4)

The supplementary diagnostic techniques comprised radiological assessments conducted on the subjects in both cohorts, systematically recorded according to anatomical region. There were no differences in the use of other radiographic techniques between the two samples, except for panoramic radiographs, which were performed significantly more often at the HSJD (66.3%) than at the MMRRH (13.3%) (*p* < 0.001). (Table 7)

**Table 7:** Diagnostic test: radiography

X-rays Site:	MMRRH (*N* = 60)		HSJD (*N* = 89)		MMRRH (*N* = 60)				HSJD (*N* = 89)		
	YES	NO	YES	NO	*p*-value	DCM (*N* = 32)	DGD (*N* = 28)	*p*-value	DCM (*N* = 38)	DGD (*N* = 51)	*p*-value^(#)^
Skull	6(10.0%)	54(90.0%)	11 (12.4%)	78(87.6%)	0.657	2 (6.3%)	4 (14.3%)	0.404 ^(4)^	7 (18.4%)	4 (7.8%)	0.121^(4)^
Spine	19(31.7%)	41(68.3%)	31 (34.8%)	58(65.2%)	0.688	7 (21.9%)	12 (42.9%)	0.081 ^(3)^	21 (55.3%)	10 (19.6%)	< 0.01*^(3)^
Limbs	30(50.0%)	30(50%)	33 (37.1%)	56(62.9%)	0.117	14 (43.8%)	16 (57.1%)	0.301 ^(3)^	19 (50.0%)	14 (27.5%)	0.03*^(3)^
Orto.	8(13.3%)	52(86.7%)	59 (66.3%	30(33.7%)	< 0.01*	5 (15.6%)	3 (10.7%)	0.712 ^(4)^	21 (56.8%)	37 (72.5%)	< 0.01*^(3)^

(*) *p*-value < 0.05: significant. Numbers next to the *p*-values show which test was used: chi-square test (3) or Fisher’s exact test (4)

Orto: Ortopantomography/panoramic x-ray. Note: same patient may present more than one x-ray

## Discussion

The present study describes and compares DOMF disorders and their possible associations with bone diseases in children from the areas of Arusha in Tanzania, East Africa, and Barcelona in Spain, southern Europe.

Rare diseases are a challenge, because the symptoms vary widely between patients with the same condition. Their low prevalence (defined by the WHO as diseases affecting 65 per 100,000 people) means there is a shortage of medical expertise and knowledge, resulting in limited research and inadequate care [29]. This is especially the case in developing countries, where patients are often excluded from healthcare systems and from the benefits of research. Relatively common symptoms can easily mask underlying rare diseases, leading to misdiagnosis and therapeutic delay. This suffering is compounded by the fact that there is often a lack of effective treatment [30].

In this study we carried out careful physical examinations and occasionally used radiological reports to obtain a clinical diagnosis of bone pathology from existing medical records. It is important to note that in most East African countries, as is characteristic of the developing world, the diagnosis of genetic diseases associated with skeletal malformations is tentative. The relative lack of advanced healthcare resources in Tanzania makes it very difficult to perform genetic testing and specific DNA analysis to confirm diagnoses [12].

Due to the complexity of bone diseases and their manifestations [22] our research design defined two subgroups of bone diseases based on closely related etiopathogenesis. The first subgroup, Disorders in Cellular Metabolism (DCM), included cellular patterning disorders such as fibrous dysplasia, craniosynostosis, congenital limb defects and neurotube defects, spina bifida, and bone metabolic disorders such as mucopolysaccharidosis and hypophosphatemic rickets. The second group, Disorders of Bone Growth/Deformity (DGD), comprised growth disorders due to matrix dysfunction such as osteogenesis imperfecta, achondroplasia, skeletal dysplasia, bone deformities in neurofibromatosis and arthrogryposis.

To explain the relationship between rare congenital bone conditions and DOMF disorders, it is necessary to study the early stages of embryological development and the trilaminar germinal disc [31]. While bone and cartilage originate from the mesoderm, teeth originate from two of the primary germ layers, the ectoderm and the mesoderm. At around week 6 of embryological development, neural crest cells interact with the ectoderm and the mesoderm at the level of the embryo’s stomodeum, or primitive mouth, to form a key differentiated structure known as the ectomesenchyme. It is from this new laminal structure that odontogenesis will begin. Indeed, this cellular contribution from the neural crest which gives rise to the ectomesenchyme lamellae will provide the material o dentin and pulp. The cementum and periodontal ligament, as well as the alveolar bone, are also derived from this lamella [31].

In addition to the cellular derivations of the trilaminar germinal disc, the two events of bone ossification, endochondral and intramembranous ossification, also illustrate the relationship. Intramembranous ossification is the direct differentiation of mesenchyme cells into osteoblasts, followed by bone matrix formation and biomineralization; examples include the formation of the skull, maxilla and mandible. For its part, endochondral ossification is the formation of bone cells from the pre-existing cartilage template for the future skeleton [13, 32]. Therefore, understanding the meeting point between bone and teeth and the pathophysiology of some bone diseases and their association with DOMF disorders in children would add to our knowledge of their clinical expression [11].

There was no significant difference in the age of patients with bone pathology in the two samples or between the DCM and DGD subgroups. The findings are consistent with those from other research, which has suggested that pre-school children represent a high percentage of patients with oral health problems, especially in areas of lower socioeconomic status and limited access to oral healthcare resources [33, 34].

Previous reports have suggested that the nutritional problems most commonly associated with skeletal dysplasia are gastrointestinal problems, dysphagia, dental problems and feeding difficulties, which may lead to reduced body weight in children. Levels of BMI above the standard cut-offs (6–8% in children and adolescents and 13% in adults) are found in disorders such as mucopolysaccharidosis, osteogenesis imperfecta and achondroplasia, which are usually associated with obesity. The most likely explanation is reduced mobility as a result of orthopedic problems, low body energy expenditure, and metabolic complications [35].

A number of studies have shown that patients diagnosed with rare bone diseases are more likely to have poor oral hygiene and dental caries [34, 36, 37]. These studies have also reported a direct correlation between limited access to oral health services and the absence of an accurate diagnosis for rare genetic diseases [36, 37]. This situation mirrors the low availability of dental services in developing countries such as Tanzania, due to the shortage of dentists, poor nutrition and limited parental awareness of the importance of oral health [34–36].

Miranda-Rius et al. reported a high prevalence of periodontal diseases and dental fluorosis in a healthy secondary school population living in a volcanic area in northern Tanzania [7]. The present study indicated a significant prevalence of dental fluorosis in patients with bone pathology at the MMRRH, and that it was more frequent in the DCM subgroup than in the DGD subgroup, regardless of severity. The fluoride content of drinking water in the Arusha region is approximately 3.6 mg/l, which significantly exceeds the WHO’s recommended level of 0.5-1 mg/l [7–9]. The extensive use of magadi salt as a food additive has been found to be strongly correlated with elevated levels of dental fluorosis (TF levels 5–9) [9]. Excess fluoride can lead to defective enamel formation, affecting the maturation of permanent dental tissue during the crucial first six years of life. Furthermore, increased fluoride levels have been linked to the exacerbation of dental caries [38]. The DCM subgroup in our study exhibited clear evidence of this effect.

In the general population, the prevalence of dental agenesis and supernumerary teeth is 3.8% and 4.5% respectively [15]. It is important to note that most dental anomalies in the MMRRH sample were recorded clinically, without radiological confirmation; by contrast, this complementary examination was performed in all patients in the HSJD samples, since it is covered by the health system in Spain. Within this context, the term ‘clinical absence of teeth’ was used as a substitute for ‘dental agenesis’. Clinical absence of teeth was found to be significantly more prevalent at the MMRRH than at the HSJD, in agreement with the findings of other authors [15]. The prevalence of supernumerary teeth was reported to be lower in the MMRRH sample, although this figure may be an underestimation since the cases were not confirmed by dental radiographs.

The current study reported a high frequency of class II and III malocclusions in both hospital samples, regardless of the subgroups analysed. These results corroborate those published by other authors [36, 37, 39–41]. Furthermore, the HSJD sample showed a significant trend towards overjet values >3 mm (especially in the DCM subgroup) while inverted overjet was more prevalent in the MMRRH sample, who also had more crossbite malocclusions. Our findings for crossbite and/or inverted overjet are consistent with other reports in osteogenesis imperfecta, fibrous dysplasia and hypophosphatemic rickets [42–44].

The vast majority of the MMRRH sample had bone pathology, with skeletal deformities most commonly found in the upper and lower limbs and spine. Lower limb deformities were significantly more frequent at the MMRRH than at the HSJD. Joint studies have reported similar skeletal manifestations of genetic disorders, but have focused on developmental or metabolic disorders or miscellaneous entities [3, 11, 45]. Easy accessibility and modern orthopedic surgical correction at the HSJD has reduced the incidence of limb deformities despite higher rates of achondroplasia, osteogenesis imperfecta and mucopolysaccharidosis in this region. The availability of various surgical correction options ranging from osteotomy to Ilizarov, limb lengthening and complex deformity surgery [46–49] contrasts with the limited access to orthopedic and neurosurgical services in Tanzania.

Unlike previous reports which have focused on a single disease entity, in this study we combined some of the etiologically related syndromes or diseases into subgroups and then compared the differences between DOMF and bone pathologies. In DGD patients, growth disorders, especially defective matrix functions such as genetic alterations in genes (e.g., COL 1 A, COLA2), or alterations in receptor formation (FGFR3) contribute to bone fragility and low density which can precipitate pathological fracture [26, 42]. In this subgroup most patients had osteogenesis imperfecta, followed by achondroplasia. Pathological fractures were significantly more prevalent in the DGD subgroup than in the DCM subgroup in both hospitals. Genetic analysis is strongly recommended in view of the clinical and genetic heterogeneity of skeletal disorders [31].

In low and middle income countries (LMICs) like Tanzania, the coverage of genetic services, including prenatal diagnosis and counselling for individuals and families to prevent congenital and genetic diseases, is extremely limited. Recently the country’s authorities have sought to improve antenatal care, with the provision of essential nutritional supplements such as folic acid and iron to prevent anemia and neurotube defects, but other genetic disorders such as those attributed to limb deformities cannot be detected or prevented. For this reason, the WHO recommends the implementation of community genetics programs in LMICs, with a focus on preventing congenital and genetic diseases at the population level. These programs should provide genetics services, including diagnosis and counselling, to individuals and families [33].

In developing countries, the possibility of obtaining a definitive diagnosis (genetic and/or molecular) in the case of uncommon diseases is practically non-existent, and health professionals are rarely able to provide sufficient information. In East Africa, patients suffering from bone disease do not have access to the sophisticated metabolic testing that could help diagnose the condition. The diseases of the skeletal system studied here have a rare clinical picture, which means that diagnosis can take years. In the meantime, carers face challenges related to the lack of specialists able to make an accurate diagnosis and the unavailability of complementary tests that could expedite the process, such as genetic and laboratory tests [50, 51].

In general, pediatric patients with rare bone diseases are usually treated by specialist teams comprising various disciplines, such as neurology, cardiology, psychiatry, orthopedics, physical therapy, ophthalmology and otolaryngology. However, dental care often plays a secondary role, or is even non-existent. This, in turn, underlying dental problems and reduces the overall quality of life in patients with diseases of the skeletal system [52]. This limited access to dental care in this population affects their oral health and decreases the likelihood of early intervention, with the result that the treatments eventually indicated tend to be more radical and costlier. The physical limitations of this group may also impact their ability to maintain adequate oral health care [53].

A notable limitation of the present study, particularly in the MMRRH sample, was the small number of participants who provided either orthopantomograms or dental radiographs. As a result, most diagnoses of dental abnormalities were clinical and were probably underreported. In East Africa, bone and oromaxillofacial disorders are treated by a small number of dentists, pediatricians and sometimes general practitioners, who are the first to see these patients due to the severe shortage of dentists. As a result, the oromaxillofacial changes associated with bone diseases are often overlooked.

In summary, certain bone diseases with the same etiology present similar dentomaxillofacial abnormalities. In this study, for example, the bone growth/deformity (DGD) group were associated with significantly higher rates of tooth agenesis, Class III malocclusion with reverse overbite and crossbite than the disorders in cellular metabolism (DCM) group. Conversely, DCM patients were found to be associated with a significantly higher prevalence of dental fluorosis and open bite malocclusion than their peers with DGD. Returning to the comparison at the level of the hospitals, the MMRRH group presented higher degrees of dental fluorosis and clinical tooth agenesis, and less dental overjet than the HSJD group [54].

This study may help to draw attention in the field of health sciences to certain rare or minority diseases in East Africa. The creation of guidelines for general practitioners, pediatricians, dentists and nurses in Tanzania would improve their ability to detect clinical signs compatible with certain bone diseases and oromaxillofacial disorders. The patients affected are particularly vulnerable from a psychological, social, economic and cultural point of view, and so improvements in this area are likely to have a notable impact on their quality of life. The burden of DOMF can be reduced and the quality of care for people with rare diseases enhanced through the establishment of collaborative programs focusing on training, research and capacity building involving these two socio-geographical areas.

## Conclusions

This study clearly shows an association between dentomaxillofacial abnormalities and bone diseases in two pediatric populations from southern Europe and East Africa. The MMRRH group exhibited significantly higher rates of gingivitis, dental fluorosis, clinical dental agenesis, and malocclusion than the HSJD group.

We conclude that the use of a mechanistic approach to classify bone diseases according to their etiopathogenesis allows us to explain certain DOMF disorders and can alert clinicians to the likely presence of these abnormalities.

## Data Availability

The datasets used and/or analysed during the current study are available from the corresponding author on request.

## Abbreviations

DCM: Disorders in cellular metabolism
DGD: Disorders of bone growth/deformity
DMF-T: Decayed, Missing, Filled Teeth index
DOMF: Dentomaxillofacial
HSJD: Hospital Sant Joan de Déu
GI: Löe & Silness Gingival Index
MMRRH: Mount Meru Regional Referral Hospital
PI: Silness & Löe Plaque Index
